# Stored object knowledge and the production of referring expressions: the case of color typicality

**DOI:** 10.3389/fpsyg.2015.00935

**Published:** 2015-07-06

**Authors:** Hans Westerbeek, Ruud Koolen, Alfons Maes

**Affiliations:** Tilburg Center for Cognition and Communication, Tilburg UniversityTilburg, Netherlands

**Keywords:** reference production, color typicality, content determination, cognitive visual saliency, models of reference production

## Abstract

When speakers describe objects with atypical properties, do they include these properties in their referring expressions, even when that is not strictly required for unique referent identification? Based on previous work, we predict that speakers mention the color of a target object more often when the object is atypically colored, compared to when it is typical. Taking literature from object recognition and visual attention into account, we further hypothesize that this behavior is proportional to the degree to which a color is atypical, and whether color is a highly diagnostic feature in the referred-to object’s identity. We investigate these expectations in two language production experiments, in which participants referred to target objects in visual contexts. In Experiment 1, we find a strong effect of color typicality: less typical colors for target objects predict higher proportions of referring expressions that include color. In Experiment 2 we manipulated objects with more complex shapes, for which color is less diagnostic, and we find that the color typicality effect is moderated by color diagnosticity: it is strongest for high-color-diagnostic objects (i.e., objects with a simple shape). These results suggest that the production of atypical color attributes results from a contrast with stored knowledge, an effect which is stronger when color is more central to object identification. Our findings offer evidence for models of reference production that incorporate general object knowledge, in order to be able to capture these effects of typicality on determining the content of referring expressions.

## Introduction

**FIGURE 1:**
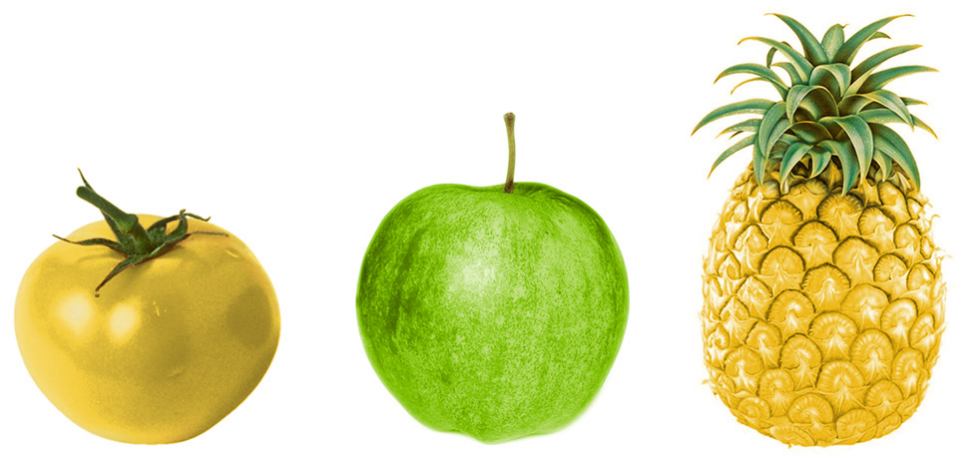
**An example of a visual context, containing an atypically colored object.** Manipulations of color may not be visible in some print versions of this paper.

In everyday language use, speakers often refer to objects by describing what they see, in such a way that an addressee can uniquely identify the intended object (e.g., Pechmann, 1989; Brennan and Clark, 1996; Horton and Gerrig, 2005; Arnold, 2008; Van Deemter et al., 2012a). In **Figure 1**, for example, a speaker can refer to the leftmost object by using the definite description “the yellow tomato.” In this visual context this referring expression accommodates unambiguous identification by the addressee, as it describes the target object and rules out the other (distractor) objects. Note, however, that a description like “the tomato” would also suffice as an unambiguous description of the leftmost object, as there are no other tomatoes in the context. Then why would a speaker mention the tomato’s color anyway?

A reason could be that the color of the yellow tomato in **Figure 1** draws attention, because it contrasts with one of the features in a stored representation of tomatoes in the speaker’s long-term memory, namely the feature that tomatoes are typically red. This makes the color of the tomato cognitively salient. Cognitive salience is different from physical salience, which is visual salience caused by image-level characteristics such as bright colors and strong contrasts (we take the terms cognitive and physical salience from Landragin, 2004). As such, the tomato’s color may not be physically different from the color of the pineapple, but when cognitively processed the color of the tomato is more conspicuous. As speakers are inclined to mention object properties that capture their attention or the attention of the addressee (e.g., Krahmer and Van Deemter, 2012), the yellow tomato’s atypical color probably causes the speaker to include this in the referring expression, even though this property may not be strictly necessary for unique identification. If speakers are influenced by atypical colors, that implies that speakers are sensitive to contrasts with stored object knowledge when they determine the content of a referring expression.

The question of content determination (i.e., which properties of an object does a speaker include in a referring expression?) is often addressed from both a psycholinguistic perspective and in the field of natural language generation (NLG). Psycholinguistics provides models of content determination by human speakers (e.g., Brennan and Clark, 1996; Engelhardt et al., 2011), for example by addressing the question whether object properties are mentioned merely because they are salient to the speakers themselves, or also because these properties may be found useful for the addressee, whose task it is to identify the referred-to object (e.g., Brennan and Clark, 1996; Horton and Keysar, 1996; Arnold, 2008). NLG models make comparable predictions on content determination, as they often aim to simulate human referring behavior (e.g., Dale and Reiter, 1995; Frank and Goodman, 2012; Krahmer and Van Deemter, 2012).

Models of reference, either implicitly or explicitly, describe at least two (addressee-oriented and speaker-internal) types of factors that speakers rely on when determining the content of a referring expression. The first is how informative an object property is for addressees: when, for example, a property is unique to an object in a context, this property is highly informative with respect to the addressees’ task to identify the target object, as it rules out all other objects in the context. As such, informativeness can be regarded as a mainly addressee-oriented factor in content determination. The other factor, salience, is essentially more speaker-internal: speakers tend to mention object properties that capture their visual attention (e.g., Conklin and McDonald, 1982; Brennan and Clark, 1996; Fukumura et al., 2010; Frank and Goodman, 2012; Krahmer and Van Deemter, 2012). This is not to say that addressees would not benefit from object properties that are included in a referring expression based on salience. Speakers’ decisions with respect to content determination may reflect addressee-oriented considerations as well (we will further elaborate on this in the general discussion).

While both informativeness for addressees and salience for speakers are part of current models of content determination in reference production, specific extensions may be needed to capture the potential effects of atypicality on content determination. Without such extensions, models of reference would not predict that atypical colors are more salient to speakers (and addressees), and thus would model referring expressions that are identical despite differences in color atypicality.

To test how atypicality may affect content determination, we focus on atypical colors, and study definite descriptions produced by speakers referring to typically and atypically colored objects. Our hypotheses are: (1) A higher proportion of descriptions will include the color of atypically colored objects, compared to typically colored ones; (2) this proportion is correlated to the degree to which a color is atypical for an object; and (3) this proportion is higher when shape is less diagnostic for the identity of an object. Our null hypothesis would be that speakers base content determination on informativeness and physical salience, and thus would not be sensitive to differences in atypicality of target objects.

### Theoretical Background

The cognitive processes that underly our predictions for effects of color atypicality on reference production are rooted in the psychology of object recognition. Object recognition is an integral part of speaker-internal processes in reference production. When speakers refer to visually perceived objects, such as the tomato in **Figure 1**, they must first recognize and identify this object as being a member of the category *tomato*. Recognizing objects implies assessing a stored representation of an object in long-term memory, which in turn yields a phonological representation of the object’s name (e.g., Humphreys et al., 1988). This will then be realized as the head noun of the referring expression. Stored knowledge of the typical colors of objects plays a role in this process of object recognition and naming.

That atypicality affects object recognition follows from work in experimental psychology (e.g., Tanaka and Presnell, 1999; Tanaka et al., 2001; Therriault et al., 2009). In several studies, it is shown that color plays a role in object recognition through response latencies for example, as people are slower to recognize and name objects that are atypically colored (e.g., Price and Humphreys, 1989; Therriault et al., 2009), or through Stroop tasks (Naor-Raz et al., 2003). These effects are caused by the fact that an atypical color cannot function as a useful cue for finding the corresponding mental representation of the object. Also, atypically colored objects are visually salient and thus likely attract attention in a scene (e.g., Becker et al., 2007). These studies show that for (at least some) objects color is part of an object’s representation in stored knowledge, and that this is accessed when objects are recognized (see Tanaka et al., 2001 and Bramão et al., 2011a, for comprehensive reviews).

Not all objects are strongly tied to one or a few particular colors. The degree to which a particular object is associated with a specific color is called color diagnosticity (e.g., Tanaka and Presnell, 1999). Objects that can have any color are called non-color-diagnostic. The color of these objects is not predictable from the object’s category (e.g., Sedivy, 2003; Bramão et al., 2011a), as theys can have many different colors (e.g., cars, pens). Conversely, objects that do have one or a few prototypical colors associated with them are called color-diagnostic objects (e.g., bananas, carrots), because color is diagnostic in determining their identity, and can be predicted from the object’s category (e.g., Tanaka and Presnell, 1999; Bramão et al., 2011a,b).

To study effects of atypicality, the focus is on color-diagnostic objects, because the color of these objects can be more or less like the prototypical color of the category the object belongs to. As said, in stored knowledge, the mental representation of such objects plausibly contains information about what their typical color is (e.g., Naor-Raz et al., 2003). This information is based on the color of objects in the same ontological category: if many exemplars of an object have the same color, then this color is prototypical of the object’s category (e.g., Rosch and Mervis, 1975). This does not rule out that other colors are possible too: Rosch’s (1975) Prototype Theory postulates that one object exemplar can simply be a better representative of the category than another. So, the exact color used is one factor that determines how atypical a color is for an object: for example, blue is very atypical for bananas, but green not so much.

Within the category of color-diagnostic objects, higher, and lower color-diagnostic objects can be distinguished (e.g., Tanaka and Presnell, 1999). For high color-diagnostic objects, color is an important feature in determining their identity. Typical examples of such objects are fruits: often a fruit’s shape is simple and similar to other fruits (i.e., round with only a few protruding parts), which makes color more diagnostic in identification (e.g., Tanaka et al., 2001). So, when other aspects of objects such as shape are more characteristic, color is likely to be less instrumental in object recognition (Rosch and Mervis, 1975; Mapelli and Behrmann, 1997; McRae et al., 2005; Bramão et al., 2011a, p. 245). Shape diagnosticity is, for object recognition, a moderating factor in the degree of association between an object and its typical and atypical colors: once viewers have to recognize atypically colored objects having a highly diagnostic shape, we may expect color to be less crucial in the recognition of the object, as the process will be informed more prominently by the diagnostic shape. It may be assumed that manipulations of color typicality are more conspicuous for objects with a relatively simple shape (e.g., lemons) than for complex-shaped objects (e.g., lobsters).

As color atypicality is important for object recognition (and more so if objects have a low-diagnostic shape), and atypical colors capture visual attention (Landragin, 2004; Becker et al., 2007), what does that mean when speakers have to produce an adequate referential expression for visually present objects? In general, speakers are inclined to mention what captures their visual attention in referring expressions, which may be useful for addressees (e.g., Conklin and McDonald, 1982; Brennan and Clark, 1996; Keysar et al., 1998; Fukumura et al., 2010; Frank and Goodman, 2012; Krahmer and Van Deemter, 2012). Hence, for physical salience, the link with content determination is indeed well-established. For example, color contrast causes speakers to mention color in their object descriptions (e.g., Viethen et al., 2012; Koolen et al., 2013). But what about cognitive salience, and color (a)typicality in particular? We expect that the cognitive salience associated with atypical colors also results in color being a highly preferred attribute when speakers have to produce adequate referential expressions for atypically colored objects.

The idea that stored knowledge of typical colors of objects plays a role in content determination gains support from a production study by Sedivy (2003). Her work does not involve atypical colors, but she investigated whether speakers mention color in a referring expression dependent on the color diagnosticity of the objects they describe. Participants gave instructions to a conversational partner to move one of two (typically) colored drawings of objects. In the experimental trials, color was not necessary for helping the addressee to disambiguate the target object from the other object, so mentioning color would yield what is called an overspecified referring expression (e.g., Pechmann, 1989; Koolen et al., 2011). The target objects (i.e., those that were to be moved) were either color-diagnostic (e.g., yellow bananas), or non-color-diagnostic (e.g., yellow cars). Sedivy (2003) observed that for color-diagnostic objects, the proportion of speakers that mentioned the (predictable) color of such objects was roughly thirty percent lower than when objects were not color-diagnostic. All objects in Sedivy’s experiment were typically colored, and it is yet unclear whether colors that contrast with stored knowledge will also make speakers include color. Sedivy’s (2003) results, however, do suggest that content determination is affected by color information in object knowledge, and that speaker’s decisions to encode color in a referring expression are not taken independently of an object’s type.

Participants in a study by Mitchell et al. (2013a) described objects with atypical materials or shapes, where mentioning these properties was necessary for the addressee to uniquely identify the intended object. Although not dealing with color, Mitchell et al.’s (2013a) study directly suggests that atypical object properties are preferred over typical ones in content determination. In their experiment, participants instructed a lab assistant to move a number of objects on a table into positions in a grid. Target objects could not be uniquely identified by mentioning their type only, so participants had to include shape, texture, or both in their referring expressions in order to be unambiguous. Crucially, Mitchell et al. (2013a) manipulated whether the shape of the object was atypical (e.g., a hexagonal mug), or whether the material was atypical (e.g., a wooden key), and using neither of those properties would result in an ambiguous referring expression. Thus, for unique identification of the target objects the speakers had to decide between mentioning a typical property, an atypical one, or both. Speakers turned out to prefer the atypical property over the typical one significantly more often than the other way around.

So, previous work on reference production in combination with color diagnosticity and typicality shows that speakers to mention atypical properties of objects when referring to them. Nonetheless, there are some ways in which this work can be extended, with respect to overspecification, effects of color diagnosticity and typicality in object recognition, and the specific use of color adjectives. Firstly, it is yet unclear whether atypicality leads speakers to mention an atypical property that is not needed to uniquely identify the target object, but will yield an overspecified referring expression instead. In Mitchell et al.’s (2013a) task, mentioning the atypical property always disambiguated the target object from distractors, and as such one can speculate that the preference of speakers for the atypical property over the typical one may not only be due to the atypicality *per se*, but also because speakers may have found the atypical property somehow more informative or useful than the typical alternative. Such decisions may be different when the atypical property is not needed to uniquely identify the object. Secondly, Mitchell et al.’s (2013a) data does not provide insight into a potential relationship between the degree of atypicality of an object property and the probability that it is included in a referring expression. It may be less straightforward to define a degree of atypicality for a shape or material given some object, but this is possible in the case of color typicality. Finally, we argue that it is interesting to look specifically at color, because color is often found to be one of the most salient properties of objects and is realized in referring expressions more often than any other property (e.g., Pechmann, 1989), also in more naturalistic domains (e.g., Mitchell et al., 2013b).

### The Current Experiments

To investigate how effects of color atypicality in object recognition may affect content determination in reference production, we test whether speakers redundantly include color in a referring expression, and whether this is proportional to the degree of (a)typicality of that color for the object that is referred to. Following the object recognition literature, the degree to which specific objects are associated with particular colors theoretically depends on two factors. One factor is the degree of color atypicality: Some colors are more atypical for an object than other colors (e.g., blue bananas are more atypical than green ones). The other factor is shape diagnosticity: manipulations of color typicality are expected to be more conspicuous for low-shape-diagnostic objects (e.g., lemons) than for high-shape diagnostic ones (e.g., lobsters), because for the former type of objects color may be less crucial in object recognition. Given the integral role of object recognition in reference production, the question is how these factors affect the production of referring expressions.

In two language production experiments, speakers view simple visual contexts comprised of multiple typically and atypically colored objects. The speakers are instructed to describe one of the objects in such a way that a conversational partner can uniquely identify this target object. The contexts are constructed as such that color is never necessary for unique identification. As such, we keep the informativeness of color for the addressees’ task to identify the intended referent equal across all conditions. So, when speakers mention color, this is in a strict sense redundant. In Experiment 1, we investigate how the degree of atypicality of a color for the target object (on a continuum, established in a pretest) affects the proportion of descriptions including color. We aim to maximize the diagnostic value of color by focusing on objects with a low-diagnostic shape (e.g., Bramão et al., 2011a). In Experiment 2, we compare typically and atypically colored objects that have a shape that is more versus less diagnostic, in order to address the second factor that is expected to moderate color typicality. So, we investigate whether our findings from the first experiment extend to objects for which color itself is a less central property, and whether shape diagnosticity moderates speaker’s sensitivity to color atypicality in reference production.

## Experiment 1: Referring to Objects with Colors of Different Degrees of Atypicality

### Method

#### Participants

Forty-three undergraduates (eleven men, thirty-two women, median age 21 years, range 18-25) participated for course credit. The participants were native speakers of Dutch (the language of the study). All gave consent to have their voice recorded during the experiment. Their participation was approved by the ethical committee of our department.

#### Materials Pretest

A pretest was conducted to determine the degree of atypicality of objects in certain colors. Sixteen high-color-diagnostic objects were selected on the basis of stimuli used in object recognition studies (e.g., Naor-Raz et al., 2003; Therriault et al., 2009). These objects were mainly fruits and vegetables, with simple shapes. In terms of geons (cf., Biederman, 1987), they were mainly comprised of one or two simple geometric components. Such simple objects have an uncharacteristic shape, as shape is relatively uninformative for distinguishing these objects from other object categories (Tanaka et al., 2001). This makes color more instrumental in object recognition (Bramão et al., 2011a). For each of the objects a high quality photograph was obtained, which was edited such that the object was on a plain white background. Further photo editing was done to make a red, blue, yellow, green, and orange version of each object. This resulted in a set of eighty photos (16 object types in five colors).

The photos were presented to forty participants in an on-line judgment task (thirteen men, twenty-seven women, median age 22.5 years, range 19-54; none participated in any of the other experiments and pretests in this paper). To manage the length of this task, participants were randomly assigned to one of two halves of the photo set. For each photo, participants first had to type in the name of the object (“what object do you see above?”) and the object’s color (“which color has the object?”). Then, they answered the question “how characteristic is this color for this object?” by using a slider control ranging from “is not characteristic” to “is characteristic” (“niet kenmerkend,” “wel kenmerkend” in Dutch). The position of the slider was linearly converted to a typicality score ranging from 0 to 100, where 100 indicated that the color-object combination was judged as most typical (i.e., the slider was placed in the rightmost position). For each photograph, the typicality score was averaged over participants in order to calculate a measure of color typicality.

#### Materials

Based on the results of the pretest, fourteen objects were selected for the experiment. Two objects were rejected because typicality scores were low for all the colors tested, or because many participants had difficulties naming the object (see the supplementary materials for details). Furthermore, of each object two colors were discarded, such that the final set of objects and colors would represent the whole spectrum of the typicality ratings continuum obtained in the pretest (scores ranging from 2 to 98, from very atypical to very typical, plus scores in between). As an illustration: the least typical objects were a blue bell pepper and red lettuce, among the most typical ones were yellow cheese and a red tomato. A yellow apple and a green tomato fell about halfway in between the extremes.

**FIGURE 2:**
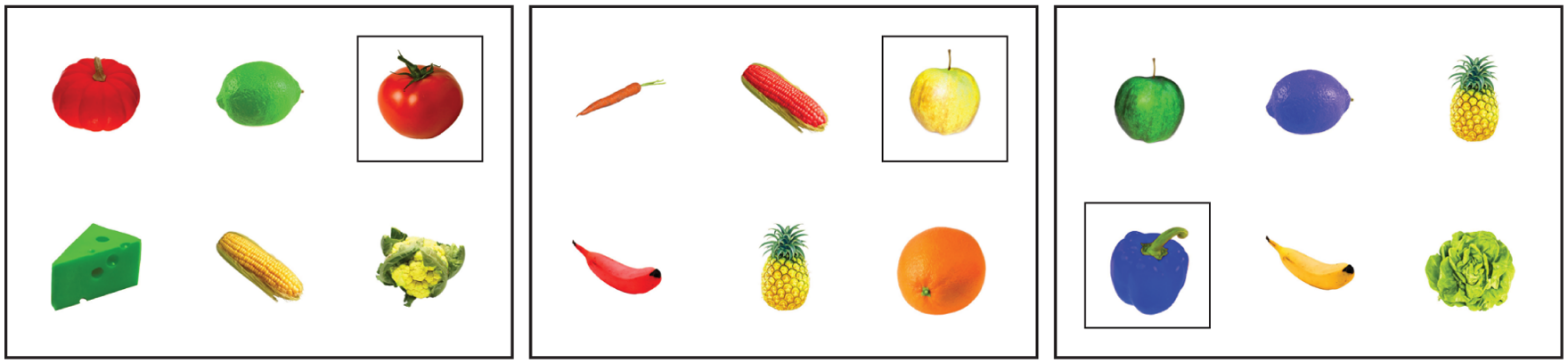
**Examples of visual contexts in Experiment 1.** From left to right: a context with a highly typical target (red tomato; typicality score 97), one with a not typical nor atypical target (yellow apple; typicality score 58), and one with an atypical target (blue pepper; typicality score 2).

The final set of objects was used to construct forty-two experimental visual contexts. **Figure 2** presents three examples of these contexts. Each context contained six different objects, positioned randomly in a three by two grid. The colors of these objects were chosen such that there were three different colors in each context, with each color appearing on two objects. Also, the typicality score averaged over the six objects in each context was similar for all trials (the mean typicality score of each context was between 40 and 60). One of the objects in each context was the target object, which was marked with a black square outline. The other five objects were the distractors. The target object was always of a unique type in each context, so mentioning the target object’s color was never necessary to disambiguate the target from any of the distractors. Crucially, the 42 target objects differed in their degree of typicality, as established in the pretest.

To ensure that the degree of color typicality of the target object was not confounded with physical salience, we assessed salience by using a computational perceptual salience estimation algorithm (Erdem and Erdem, 2013). We did this because any effect of color atypicality on whether speakers mention color in a referring expression should not be attributable to the object’s color being more bright, contrasting, or otherwise physically salient to the speaker. Crucially, the algorithm that we used does not incorporate any general knowledge about objects and their typical colors, as it only measures salience based on physical (image-level) features.

We ran Erdem and Erdem’s (2013) algorithm on our 42 experimental visual contexts, using its standard settings and parameters. The algorithm outputs physical salience scores for each pixel of an image, which expresses the relative salience of that pixel with respect to other pixels in the image. In our visual contexts, six areas of interest (AOIs) were defined, one for the target object and five for the distractor objects. Of each AOI, the mean relative salience of the pixels was calculated, which expresses how salient the object in that AOI is compared to the other AOIs (i.e., objects) in the context.

Analyses of the mean relative salience as determined by the algorithm showed that there was no significant correlation between the degree of physical salience of the target object in each scene and its color typicality, Pearson *r*(40) = 0.05, *p* = 0.721. The atypically colored objects in our experiment were physically not more salient than the typically colored ones (and vice versa). Furthermore, a one-way analysis of variance with color as the independent and salience as the dependent variable showed no differences in salience for each of the five target colors, *F*(4,41) = 1.05, *p* = 0.397.

In addition to the experimental contexts, we created 42 filler contexts. These consisted of four hard-to-describe greebles (Gauthier and Tarr, 1997), all purple, so that participants were not primed with using color in the other trials. One greeble was marked as the target object that had to be distinguished from the distractors.

#### Procedure

Participants sat at a table facing the experimenter, with a laptop in front of them. The participants were presented with the 42 trials, one by one, on the laptop’s screen. Between each experimental trial, there was a filler trial. Participants described the target objects in such a way that the experimenter would be able to uniquely identify them in a paper booklet. The instructions emphasized that it would not make sense to include location information in the descriptions, as the addressee would see the objects in a different configuration. Participants could take as much time as needed to describe the target, and their descriptions were recorded with a microphone. The addressee (experimenter) never asked the participants for clarification, so the data presented here are one-shot references.

The procedure commenced with two practice trials: one with six non-color-diagnostic objects in different colors, and one practice trial with greebles. Once the target was identified, this was communicated to the participant, and the experimented pressed a button to advance to the next trial. The trials were presented in a fixed random order (with one filler after each experimental trial). This order was reversed for half of the participants, to counterbalance any potential order effects. After completion of the experiment, none of the participants indicated that they had been aware of the goal of the study. The experiment had an average running time of about 25 min.

#### Research Design and Data Analysis

For each of the experimental trials, we determined whether the speakers’ description of the target object resulted in unambiguous reference, which mainly implied annotating whether respondents used the correct type attribute. Because the target object was always of a unique type in each context, mentioning type was sufficient. We also assessed whether the object’s type was named correctly. Using the correct type was important, because otherwise we could not deduce whether the object’s color was regarded as typical or atypical. We annotated each description as either containing a color adjective, or not.

Whether mentioning color was related to the degree of color atypicality of the target object was analyzed using logit mixed models (Jaeger, 2008). Initial analyses revealed that stimulus order had no effects, so this was left out in the following analyses. In our model, color typicality (as scores on the pretest) was included as a fixed factor, standardized to reduce collinearity and to increase comparability with Experiment 2. Participants and target object types were included as random factors. The model had a maximal random effect structure: random intercepts and random slopes were included for all within-participant and within-item factors, to ensure optimal generalizability (Barr et al., 2013). Specifically, the model contained random intercepts for participants and target objects, and a random slope for color typicality at the participant level.

### Results and Discussion

The data of three participants was not analyzed because of technical issues with the audio recordings. Of the remaining 1680 descriptions, 1629 descriptions (97%) were intelligible, unambiguous, and contained a correct type attribute, resulting in unique reference. As expected, practically all analyzed descriptions were of the form “the tomato” or “the yellow tomato.”

**FIGURE 3:**
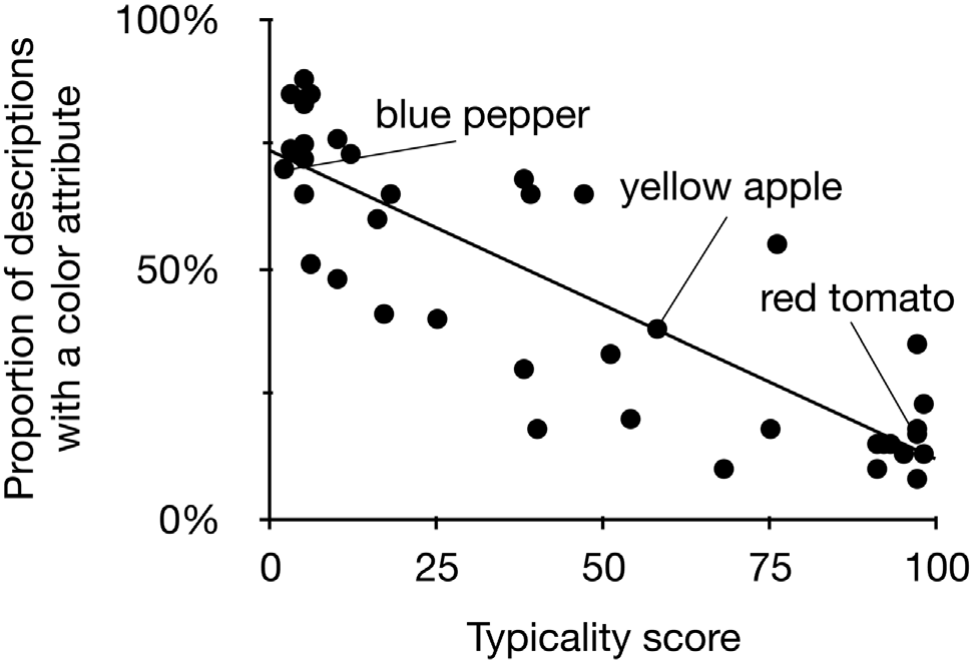
**Typicality scores of objects (horizontal axis) and the proportion of descriptions of these objects that contain color (vertical axis) in Experiment 1.** Some illustrative objects are labeled in this plot; the line represents the correlation between the two variables.

**Figure 3** plots the atypicality score of a target object in the pretest against the proportion of descriptions that contained color in the production experiment (exact proportions and typicality scores are listed in the Supplementary Materials). The mixed model revealed a significant effect of color typicality on whether a target description contained a color attribute or not, β = -2.36, *SE* = 0.25, *p* < 0.001. The direction of the effect indicated that lower typicality in the pretest was associated with more referring expressions containing color. An additional analysis by means of bivariate correlation between the typicality score of each object and the proportion of speakers mentioning color for this object reconfirmed that these were significantly related, Pearson *r*(40) = -0.86, *p* < 0.001.

The results of our experiment warrant the conclusion that content determination is affected by the degree of typicality of a target object’s color. When a color is more atypical for an object, the proportion of referring expressions that include that property increases. This effect is very strong, as exemplified by the high correlation between the two variables. **Figure 3** also suggests that it is highly consistent across speakers: for a considerable number of typically colored stimuli, the percentage of speakers not using color approaches zero, and conversely, for some atypically colored stimuli this percentage approaches 100%. This supports the theory that speakers evaluate contrasts with stored knowledge about typical features of objects in long term memory when producing a referring expression.

In Experiment 1, we have manipulated the degree of atypicality of the target objects by using different colors for objects, such that the object-color combinations span a range of atypicality scores. For example, speakers have described blue tomatoes (very atypical), green tomatoes (not atypical nor typical), and red tomatoes (very typical). However, target objects in Experiment 1 were predominantly simply shaped fruits and vegetables, i.e., objects for which color is especially instrumental in their identification (as their shape is not very informative about the identity of the objects; Tanaka and Presnell, 1999; Bramão et al., 2011a). As explained in the theoretical background, the diagnostic value of an object’s color in recognition is lower when its shape is more diagnostic (Bramão et al., 2011a). Accordingly, would color atypicality be less conspicuous when shape is more diagnostic, resulting in a moderation of the color atypicality effect on reference production? Therefore, the goal of Experiment 2 is to investigate the effect of color typicality on reference production, as a function of objects’ shape diagnosticity.

## Experiment 2: Referring to Typically and Atypically Colored Objects with High or Low Shape Diagnosticity

In Experiment 2, we cross color typicality with shape diagnosticity in a language production task similar to the one used in Experiment 1. As such, we aim to extend our findings from the first experiment to low-color-diagnostic objects (with more diagnostic shapes). We expect to find a similar relationship between color typicality and content determination as in Experiment 1, but because for low-color-diagnostic objects color is less instrumental in their identification we predict that higher shape diagnosticity overall decreases the proportion of referring expressions that include color. Secondly, we predict that shape diagnosticity and color typicality interact, such that effects of color typicality are larger when shapes are less diagnostic compared to when shapes are more diagnostic.

### Method

#### Participants

Sixty-two undergraduates participated for course credit. They participated in dyads, with one participant acting as the speaker and the other as addressee. So, there were 31 speakers (7 men, 24 women, median age 22 years, range 18-25), all were native speakers of Dutch (the language of the study). None of the participants took part in any of the other experiments and pretests in this paper. They gave consent to have their voice recorded during the experiment. Their participation was approved by the ethical committee of our department.

#### Materials

High quality white-background photos of 16 target objects were selected and edited, similar to Experiment 1, and supplemented by stimuli used in object recognition studies. The typical color of these objects was either red, green, yellow, or orange. Even though the saliency algorithm we employed showed no differences in physical salience between the five target colors used in Experiment 1, we decided for Experiment 2 to not use blue objects (which were all atypical in Experiment 1), and to equally balance color frequencies throughout the experiment. As such, the proportions of target objects in each color was kept identical in all conditions.

Half of the objects were low in shape diagnosticity: they had relatively simple shapes, as they were mostly round with very few protruding parts, like in Experiment 1. The other objects were high in shape diagnosticity, having relatively complex shapes, comprising many protruding parts and no basic round shape (i.e., comprised of many geons). Such objects (e.g., lobster; see the supplementary materials for a complete list of objects used) thus have a more characteristic (diagnostic) shape, which sets it apart from other object categories.

As in Experiment 1, the target objects were placed in visual contexts of six objects. Again, the colors of these objects were chosen such that there were three different colors in each context, with each color appearing on two objects. Three of the objects were typically colored, the other three atypically colored. One of the objects in each context was the target object, singled out by a black square outline for the speaker. The other five objects were the distractors. The target object was always of a unique type, so that mentioning the target object’s color was never necessary to disambiguate the target from any of the distractors.

**FIGURE 4:**
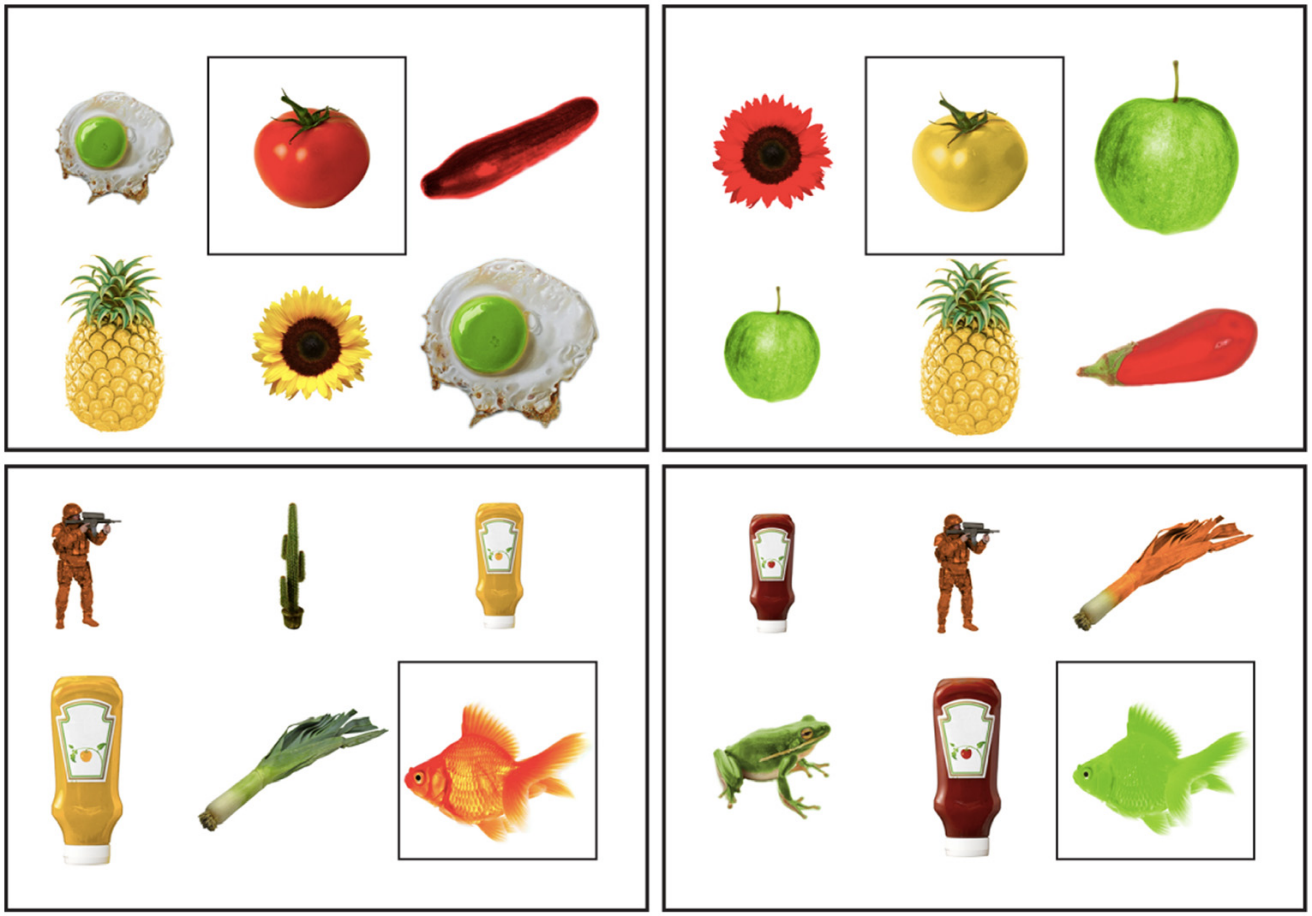
**Examples of visual contexts in each of the conditions in Experiment 2, in two color typicality conditions (horizontal axis) and in two shape diagnosticity conditions (vertical axis)**.

Eight contexts contained objects that were low in shape diagnosticity, and the other eight contexts contained objects high in shape diagnosticity. Also, in half of the contexts the target object was typically colored, and in the other half it was atypically colored. **Figure 4** presents examples of the contexts in each of the four resulting conditions: the contexts on the left contain a typically colored target object; in the contexts on the right the target has an atypical color. The upper contexts comprised of low shape diagnostic objects; the lower contexts has high shape diagnosticity.

The target objects were subjected to an on-line judgment task similar to the pretest in Experiment 1. Sixteen participants took part in this task (6 men, 10 women, median age 21 years, range 18-26; none participated in any of the other experiments and pretests in this paper). As expected, typically colored objects yielded a higher typicality score (range 87.50-99.75) than atypically colored objects range 0.83-10.50). There were no differences in typicality scores for object with a high and a low shape diagnosticity (*F* < 1), and the two factors did not interact (*F* < 1). The pretest also showed that none of the objects were difficult to name.

As in Experiment 1, we used the computational physical salience estimation of Erdem and Erdem (2013) to ensure that color typicality was not confounded with differences in relative physical salience between typical and atypical objects, and between objects with high and low shape diagnosticity. Analyses of variance of the mean relative salience of the target objects showed no differences between typically colored and atypically colored target objects (*F* < 1), nor between objects with high and low shape diagnosticity (*F* < 1). The two factors did not interact (*F* < 1). This shows that possible (interaction) effects involving shape diagnosticity cannot be ascribed to colors being physically more salient when for example shapes are simple and colored areas may appear to be larger.

#### Procedure

Participants took part in pairs. Who was going to act as the speaker and who as the addressee was decided by rolling a dice. In contrast to Experiment 1, addressees were naive participants instead of a confederate, in order to improve ecological validity (cf. Kuhlen and Brennan, 2013). Participants were seated opposite each other at a table, and each had their own computer screen. The screens were positioned in such a way that the face of either participant was not obstructed (ensuring that eye contact was possible), while participants could not see each other’s screen.

Each speaker described the target object of the sixteen visual contexts, as well as 32 filler contexts containing purple greebles. We made two lists containing the same critical trials, but with reversed typicality: target objects that were typically colored for one speaker were atypically colored for another. As such, color typicality and shape diagnosticity were manipulated within participants, while ensuring that each target object appeared in only one typicality condition for each participant. We did this because one could speculate that the overall proportion of color adjectives in Experiment 1 might inflate because participants used them to express contrasts between objects of the same type over trials. The order of the contexts in each list was randomized for each participant, but there were always two filler trials between experimental ones (i.e., one more than in Experiment 1, to further assure that that the colorful nature of our stimuli does not boost the overall probability that color was mentioned; see Koolen et al., 2013).

The addressee was presented with the same contexts as the speaker, but without any marking of the target object. Also, the objects on the addressee’s screen were in a different spatial configuration than on the speaker’s screen, in line with the instruction that it would not make sense for the speaker to mention location information. In each trial, the addressee marked the picture that he or she thought the speaker was describing on an answering sheet. Although the addressee was instructed that clarifications could be asked, there were no such requests during the whole experiment, so the data presented here are one-shot references.

The procedure commenced with two practice trials with greebles, plus one practice trial with non-color-diagnostic objects (as in Experiment 1). Once the addressee had identified a target, this was communicated to the speaker, and a button was pressed to advance to the next trial. The experiment finished when all trials were described and the addressee identified the last target object. The experiment had an average running time of about 15 min.

#### Research Design and Data Analysis

Data annotation was identical to Experiment 1. We analyzed whether using a color adjective or not was related to the degree of color atypicality of the target object using logit mixed models (Jaeger, 2008). Initial analyses revealed that stimulus list and stimulus order (trial number) had no effects, so these factors were left out in the following analyses. In our model, color atypicality and shape diagnosticity were included as fixed binomial factors, standardized to reduce collinearity and to increase comparability with Experiment 1. Participants and target object types were included as random factors. The model had a maximal random effect structure: random intercepts and random slopes were included for all within-participant and within-item factors, to ensure optimal generalizability (Barr et al., 2013). Specifically, the model contained random intercepts for participants and target objects, random slopes for color atypicality and shape diagnosticity at the participant level, and a random slope for color atypicality at the target object level.

### Results and Discussion

In total, 496 target descriptions were recorded in the experiment. 472 descriptions (95%) were intelligible, unambiguous, and contained a correct type attribute, resulting in unique reference. Practically all analyzed descriptions were of the same form as those in Experiment 1.

**FIGURE 5:**
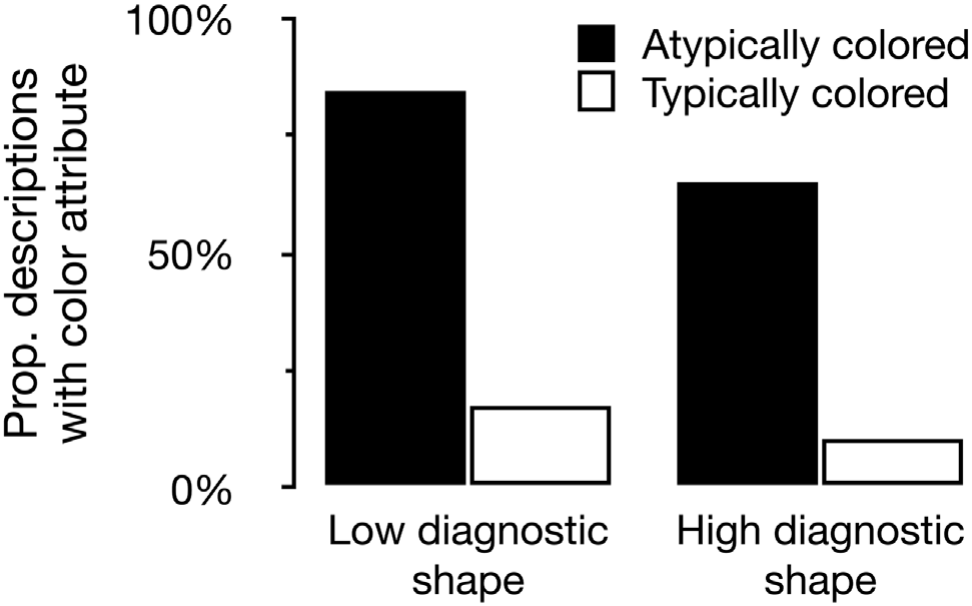
**The proportion of referring expressions containing color for each of the four conditions in Experiment 2**.

Our model revealed a significant effect of color atypicality on whether a target description contained a color attribute or not, β = 3.53, *SE* = 0.39, *p* < 0.001. Of the references to atypically colored target objects, 75.3% contained color, compared to 14.3% for typically colored target objects. Also, the model showed a significant main effect of shape diagnosticity, β = -0.89, *SE* = 0.35, *p* = 0.010. References to objects with a high diagnostic (i.e., complex) shape contained color in 38.4% of the cases, compared to 49.1% for low diagnostic (i.e., simple) shape target objects. Color typicality and shape diagnosticity interacted, such that the effect of typicality on using color in a referring expression was larger for low shape diagnostic objects than for the high shape diagnostic objects, β = -0.70, *SE* = 0.32, *p* = 0.030. **Figure 5** plots the proportion of referring expressions containing color for each of the four conditions in the experiment.

With respect to the effect of color typicality on content determination, inspection of the data revealed that not a single speaker acted against the general pattern and mentioned color more often for typically colored objects than for atypically colored ones. However, a mere three speakers mentioned color in all atypical trials, and never mentioned color in the typical trials. While most speakers showed more variation in their response to color atypicality, only these three speakers show what is often called *deterministic behavior* in the literature (e.g., Van Deemter et al., 2012b).

Experiment 2 shows that the effect of color typicality on content determination is moderated by the diagnosticity of an object’s shape. Color is more often mentioned for objects with low shape diagnosticity. It is for these objects that the color atypicality effect is slightly larger compared to objects with higher shape diagnosticity. This further supports the idea that object recognition and the status of features of objects in long-term memory is closely related to reference production.

## General Discussion

We investigated the role of speakers’ stored knowledge about objects when producing referring expression. The experiments reported in this paper show a strong effect of color atypicality on the object properties mentioned by speakers. Speakers mention the color of atypically colored objects significantly more often than when objects are typically colored, and this effect is moderated by the degree of atypicality of the color, and the diagnosticity of the object’s shape. These results support the view that stored knowledge about referred-to objects influences content determination. When a property of an encountered object contrasts with this knowledge, the probability that this property is included in a referring expression increases significantly. This also suggests that because object recognition is an integral part of reference production, there may be a close relation between findings in object recognition related to color diagnosticity and typicality on the one hand, and effects on reference production on the other.

Combined with the findings of Mitchell et al. (2013a), who report similar effects of atypical materials and atypical shapes on content determination, the current paper forms converging evidence for sizable effects of atypicality on the production of referring expressions. Furthermore, our results corroborate Sedivy’s (2003) finding that object knowledge affects content determination, and that speakers’ decisions to encode color in a referring expression are not taken independently of the object’s type. Our research also resonates with Viethen et al.’s (2012) findings on how the specific color of an object can affect a speaker’s decision to include this color in a referring expression. While Viethen et al.’s (2012). focus on colors that are relatively easy to name or not (e.g., blue versus light blue), we report effects of specific colors combined with specific object types.

We attribute the effects of color atypicality on content determination reported in this paper to the speakers’ visual attention allocation, and cognitive salience in particular: because atypical colors attract visual attention (e.g., Becker et al., 2007), speakers tend to encode these colors in a referring expression (e.g., Krahmer and Van Deemter, 2012). In the visual contexts that we used, mentioning the type of the object was always sufficient to fully disambiguate the target object from all the distractors. The speakers’ decision to include color is in that sense redundant (i.e., the referring expressions containing color are overspecified; cf. Pechmann, 1989; Koolen et al., 2011). Instead of carefully assessing the objects and their properties in the visual context, and calculating their informativeness, speakers in our experiments appeared to use other rules or mechanisms to determine the content of a referring expression.

The idea that speakers may rely on different content determination processes than calculations of informativeness has been postulated in a number of recent papers (e.g., Dale and Viethen, 2009; Van Deemter et al., 2012b; Viethen et al., 2012, 2014; Koolen et al., 2013). Instead of a careful consideration of the properties and salience of all (or a subset of) the objects in a visual context, speakers may turn to quicker, simple decision rules to make judgments in the content determination process. Such a decision rule that would fit our data would be: “If the contrast between the color of the target object and stored knowledge is strong, increase the probability that it is mentioned.”

Speakers’ reliance on relatively simple decision rules is argued to be related to the visual complexity of the contexts that they are confronted with. Some researchers hypothesize that speakers may especially rely on the “fast and frugal heuristics” in cases where considering all properties of all objects in a context is cognitively costly (e.g., Van Deemter et al., 2012b, p. 179). However, the contexts in our experiments are undoubtedly very simple: speakers only have to consider the type of six objects that are presented in an uncluttered and simple environment, which is a task that is arguably well within the speakers information processing capacity (e.g., Miller, 1956). Yet speakers seem to apply (a variation of) the aforementioned decision rule in contexts with an atypically colored target. Such contexts are not more complex or visually cluttered than the typical ones. So, the decision rule that we propose above would not be one that merely applies when the (limited) processing capacity of speakers is exceeded, but one that is universally available whenever the content of a referring expression is determined.

### Implications for (Computational) Models of Reference Production

Being able to refer to objects in a human-like manner is an important goal for NLG models of reference production (REG algorithms), and for the field of NLG (a subfield of Artificial Intelligence) in general (Dale and Viethen, 2009; Frank and Goodman, 2012; Van Deemter et al., 2012b). Our findings pose a new challenge for current REG algorithms. In the light of our findings, models can be enhanced by incorporating general object knowledge, because without access to such information they are unable to distinguish between typical and atypical objects when determining the content of a referring expression. Moreover, in our data, the decision to include color in a referring expression appears not to be taken independently of the target object’s type. For example, speakers decide to mention redness when they describe a lemon, but not when they describe a tomato. This is something that a model should be able to take into consideration.

Popular NLG models predict color use irrespective of the typicality and diagnosticity of the target’s color. In the Incremental Algorithm (IA; Dale and Reiter, 1995), attributes like color, size, and orientation are included in a referring expression on the basis of how informative they are, and they are considered one by one (i.e., incrementally). More salient attributes, like color, are considered early, because they are highly ranked in a predefined preference order (which is typically determined on the basis of empirical data). Type is likely to be included anyway, because it is necessary to create a proper noun phrase, and this would yield fully distinguishing referring expressions in all conditions in our experiments. The IA would therefore generate no color adjectives. If the IA was to be able to make the decision to mention the color of a yellow tomato, for example, and not for a red tomato, it would need a ranking (preference order) of certain colors for tomatoes (e.g., red, green, orange, yellow, blue), instead of a mere ranking of certain attributes (e.g., color, size, orientation).

The model of pragmatic reasoning by Frank and Goodman (2012) allows salience of objects to be modeled for each visual context individually (instead of in a predefined preference order). So, in effect, the salience of atypically colored objects can be modeled to be different from the salience of typically colored ones. However, Frank and Goodman (2012) calculate this (prior) salience on the basis of empirical findings, so behavioral data is needed before reference production is modeled. And while it is well possible to estimate visual salience computationally and automatically (e.g., Erdem and Erdem, 2013), such salience estimations are not (yet) able to take general knowledge into account and thus respond differently to various degrees of atypicality.

The challenge is to feed such salience estimations with knowledge about what prototypical colors of objects are, and how important color is in the identity of these objects. Assuming that object types are readily recognized computationally in a visual context (which works quite well in controlled environments nowadays, Andreopoulos and Tsotsos, 2013), a knowledge base containing prototypical object information can be queried at runtime when a referring expression is generated. This is what Mitchell et al. (2013a) and Mitchell (2013) propose in their discussion of repercussions of atypicality for REG. However, for color, a simpler system without a dedicated knowledge base may be effective too. A web search for images (e.g., on Google Images) may inform an algorithm about color typicality: when the dominant color of the first n image results of a web search is computationally determined, the prototypical color of an object should be derivable. In fact, we expect that this method can even generate the degree of atypicality of a color, much alike the typicality scores that we obtained in a pretest for Experiment 1. A comparison between the n search results showing one color and the n results showing other colors probably yields a good estimation of the degree of atypicality of that particular color.

Our results are also interesting in the light of an observed tendency toward using more naturalistic stimuli in behavioral experiments that are aimed at evaluating computational models of reference production (e.g., Coco and Keller, 2012; Viethen et al., 2012; Clarke et al., 2013; Mitchell, 2013; Mitchell et al., 2013a,b; Koolen et al., 2014). Color typicality may be an important difference between artificial and more naturalistic stimuli, as studies that employ artificial contexts often present speakers with atypically colored objects (e.g., green television sets and blue penguins; Koolen et al., 2013; Viethen et al., 2014). Our results seem to argue against using artificial contexts in reference production studies by showing that content determination can be steadily affected by atypical colors.

### Color Atypicality and Speaker-Addressee Perspectives in Reference Production

In our experiments, speakers produced referring expressions for an addressee who was present in the communicative setting. Although speakers in our experiments presumably mention the color of atypically colored target objects because atypical colors are cognitively salient to the speakers themselves, this does not necessarily assert that mentioning atypical colors more often than typical ones is exclusively speaker-internal behavior (e.g., Keysar et al., 1998; Wardlow Lane et al., 2006; Arnold, 2008). Speakers’ decisions to include color may as well be addressee-oriented and reflect what is called *audience design* in the literature (e.g., Clark, 1996; Horton and Keysar, 1996; Arnold, 2008; Fukumura and van Gompel, 2012). As suggested in the general introduction, if speakers take the addressee’s perspective into account and use their own perception as a proxy for the addressees’ (e.g., Pickering and Garrod, 2004; Gann and Barr, 2014), they may decide to mention the color of an atypically colored object because this is salient to the addressees as well.

Although the face-to-face tasks in our experiments do not offer conclusive evidence in this discussion, there are reasons to believe that overspecified atypical color attributes are beneficial for addressees. For example, a visual world study by Huettig and Altmann (2011; Experiment 3) suggests that listeners tend to look for objects in typical colors when this color is not specified for them. When listeners hear a word that refers to an object with a prototypical color (even though this color is not mentioned), their visual attention shifts toward objects that have this particular color. So, listeners likely benefit from color being included in a referring expression when this color is not in line with their expectations about the object they search for. Similar suggestions come from work in visual search, which gives reasons to assume that listeners who are informed about specific details of the target, such as its color, find the target more efficiently in real-world scenes (e.g., Malcolm and Henderson, 2009, 2010).

The addressed literature is less clear on how the interaction with shape diagnosticity that we report in Experiment 2 might translate to effects for addressees. As shape diagnosticity moderates effects of color atypicality on reference production, one could speculate that a similar moderation applies to the addressees’ task of identifying the intended target object. The object recognition literature suggests that color is relatively less instrumental in recognition for complex-shaped objects (e.g., Tanaka and Presnell, 1999; Bramão et al., 2011a), so for these objects listeners can rely more on shape-based cues in their visual search for the intended target object. Conversely, for simple-shaped objects color is a relatively more useful cue for finding these objects in a visual context (i.e., color is particularly instrumental to find the target in visual search). For example, when addressees search for a tomato, redness is a more relevant cue compared to when they search for a lobster. From this it follows (speculatively) that being informed about the color of the target object being atypical is more beneficial for listeners when they search for simply shaped objects, compared to when they search for objects for which shape is more instrumental for identifying the target. More research is needed to explore the effects of mentioning color on visual search, and interactions with color typicality and shape diagnosticity.

## Conflict of Interest Statement

The authors declare that the research was conducted in the absence of any commercial or financial relationships that could be construed as a potential conflict of interest.
