# Supplementary material for: Stored object knowledge and the production of referring expressions: the case of color typicality
Source: Front Psychol. 2015 Jul 6;6:935. doi: 10.3389/fpsyg.2015.00935 (PMC4491598; doi:10.3389/fpsyg.2015.00935)
Supplement: Supplementary file 1 [file Data_Sheet_1.PDF]

Supplementary Material

Stored object knowledge and the production of referring expressions: The case of color typicality

Hans Westerbeek[1]*, Ruud Koolen[1], Alfons Maes[1]

[1]Tilburg center for Cognition and Communication, Tilburg University, Tilburg, The Netherlands

*** Correspondence:** Hans Westerbeek, Tilburg University, room D412, PO Box 90153, 5000 LE Tilburg, The Netherlands
h.g.w.westerbeek@tilburguniversity.edu

Supplement – target objects in Experiment 1

Typicality scores for all objects in five colors as used in Experiment 1 are listed below.

Object	Typicality score per color				
	Blue	Yellow	Green	Orange	Red
**Bell Pepper**	2	91	88	76	97
**Apple**	5	58	60	92	93
**Banana**	19	91	37	25	6
**Carrot**	13	18	14	98	6
**Cheese**	3	98	5	51	9
**Corn**	9	97	19	38	5
**Grapes**	16	57	97	17	17
**Lemon**	7	95	5	71	5
**Lettuce**	3	67	92	4	3
**Orange**	13	47	19	91	10
**Pear**	5	40	68	33	18
**Pineapple**	12	75	10	54	18
**Pumpkin**	2	39	12	98	21
**Tomato**	3	21	38	65	97

In Experiment 1, initial target objects included a cauliflower and a pomegranate. After pretests the cauliflower was rejected because typicality scores were disproportionally low (typicality scores were lower than 32 for all colors). The pomegranate was rejected because many participants had difficulties naming the object (this was the case in all colors).

Supplement – results of Experiment 1

The proportion of descriptions that contained color for each target object in Experiment 1 is listed below.

Object	Color	Typicality score	Proportion of descriptions with a color attribute
**Cheese**	Yellow	98	.13
**Pumpkin**	Orange	98	.23
**Carrot**	Orange	98	.13
**Grapes**	Green	97	.18
**Corn**	Yellow	97	.08
**Bell Pepper**	Red	97	.35
**Tomato**	Red	97	.17
**Lemon**	Yellow	95	.13
**Apple**	Red	93	.15
**Lettuce**	Green	92	.15
**Banana**	Yellow	91	.15
**Orange**	Orange	91	.10
**Bell Pepper**	Orange	76	.55
**Pineapple**	Yellow	75	.18
**Pear**	Green	68	.10
**Apple**	Yellow	58	.38
**Pineapple**	Orange	54	.20
**Cheese**	Orange	51	.33
**Orange**	Yellow	47	.65
**Pear**	Yellow	40	.18
**Pumpkin**	Yellow	39	.65
**Corn**	Orange	38	.30
**Tomato**	Green	38	.68
**Banana**	Orange	25	.40
**Carrot**	Yellow	18	.65
**Grapes**	Red	17	.41
**Grapes**	Blue	16	.60
**Pumpkin**	Green	12	.73
**Pineapple**	Green	10	.48
**Orange**	Red	10	.76
**Banana**	Red	6	.85
**Carrot**	Red	6	.51
**Apple**	Blue	5	.88
**Lemon**	Green	5	.72
**Lemon**	Red	5	.84
**Cheese**	Green	5	.75
**Corn**	Red	5	.65
**Pear**	Blue	5	.83
**Lettuce**	Orange	4	.73
**Lettuce**	Red	3	.74
**Tomato**	Blue	3	.85
**Bell Pepper**	Blue	2	.70

Supplement – target objects in Experiment 2

The target objects in both shape diagnosticity conditions, and their colors and typicality scores in both color typicality conditions, as used in Experiment 2, are listed below.

Shape diagnosticity	Object	Typically colored condition		Atypically colored condition	
		Color	Typicality score	Color	Typicality score
Low (simple shape)	Basketball	Orange	95	Green	6
	Lemon	Yellow	99	Red	2
	Lettuce	Green	98	Yellow	3
	Orange	Orange	100	Green	2
	Strawberry	Red	98	Orange	7
	Tennis ball	Yellow	88	Red	8
	Tomato	Red	97	Yellow	7
	Watermelon	Green	89	Orange	2
High (complex shape)	Broccoli	Green	97	Orange	2
	Carrot	Orange	99	Red	1
	Cheese	Yellow	98	Red	1
	Chick	Yellow	95	Green	2
	Crocodile	Green	88	Orange	7
	Goldfish	Orange	95	Green	11
	Lobster	Red	91	Yellow	1
	Phone booth	Red	98	Yellow	8

Supplement – instructions for participants

Instructions for the speaker:

Thank you for participating in this research.

In this study you will get to see a number of objects on the screen. Every time, one of these objects is indicated by a square. Your task is to describe this object in such a way that the listener across the table can easily pick out the correct object from his/her screen. On that screen the same pictures will be shown as on your screen, but of course without a square, and not necessarily in the same order (you can not, and are not allowed to, use descriptions such as "the third picture from the right in the top row"). The listener writes down which picture you described.

Your descriptions will be recorded with the microphone of a headset. When the experiment commences, put the headphone on in such a way that the microphone is located around your chin. When the listener has chosen a picture, the experimenter will advance to the next series of objects. It is not intended that you enter into a conversation: you give the instructions and the listener chooses an object. You may ask whether he/she has understood you, or needs more information. You are allowed to gesture, by the way.

By signing this form you grant permission to make recordings of your voice during the course of this experiment. These recordings are processed anonymously, and will only be used for research purposes. They will never be made publicly available.

Before the experiment commences we practice with two series of objects.

If you have any questions, please ask them now. You can also ask questions after the two practice trials.

Now put the headset on and make sure the microphone is positioned correctly.

Instructions for the addressee:

Thank you for participating in this research.

In this study you will get to see a number of objects on the screen. Every time, he speaker across the table will descibe one of these objects. Your task is to write down on the back of this sheet which object you think is being described.

Sometimes you have to choose between four objects, and sometimes betwee six. Each objects is marked on the screen with a letter (A to D, or A to F). On the back of this sheet you indicate which object you think the speaker has described.

When you have chosen a picture, the experimenter will advance to the next series of objects. It is not intended that you enter into a conversation: the speaker gives the instructions and you choose an object. You may ask for more information, or ask the speaker to repeat what was just said. You are

now allowed to tell between which two objects you are in doubt about, and which specific information you need to make a decision. That is for the speaker to come up with.

Before the experiment commences we practice with two series of objects.

If you have any questions, please ask them now. You can also ask questions after the two practice trials.

(Note: These instructions are translated from Dutch.)
